# Evaluation of proprioception in patients who underwent ACL reconstruction: measurement in functional position

**DOI:** 10.3906/sag-2004-110

**Published:** 2021-08-30

**Authors:** Sinem SUNER KEKLİK, Nevin GÜZEL, Gamze ÇOBANOĞLU, Nihan KAFA, Muhammet Baybars ATAOĞLU, Zekeriya ÖZTEMÜR

**Affiliations:** 1 Department of Physiotherapy and Rehabilitation, Faculty of Health Sciences, Sivas Cumhuriyet University, Sivas Turkey; 2 Department of Physiotherapy and Rehabilitation, Faculty of Health Sciences, Gazi University, Ankara Turkey; 3 Department of Orthopedics and Traumatology, Faculty of Medicine, Gazi University, Ankara Turkey; 4 Department of Orthopedics and Traumatology, Faculty of Medicine, Sivas Cumhuriyet University, Sivas Turkey

**Keywords:** Anterior cruciate ligament reconstruction, proprioception, inclinometer, closed kinetic chain position

## Abstract

**Background/aim:**

Anterior cruciate ligament (ACL) injuries cause mechanoreceptor loss in the joint; therefore, proprioceptive deficits are observed after injury. In particular, proprioceptive measurements made in the functional position give more accurate results, and this is an area that requires further studies. This study aimed to evaluate proprioception in patients who had undergone ACL reconstruction (ACLR) in functional positions used in daily life (closed kinetic chain position), according to joint angles where ACL injuries occur more frequently, in comparison with healthy controls.

**Materials and methods:**

Thirty-four participants who underwent ACLR using a hamstring tendon graft (aged 29.18 ± 8.16 years; body mass index (BMI), 26.58 ± 4.02 kg/cm^2^) and 31 healthy participants (aged 27.35 ± 5.74 years; BMI, 24.76 ± 2.98 kg/cm^2^) were included. Proprioception was assessed with an active angle repetition test, using an inclinometer in the closed kinetic chain position while standing. Participants were asked to perform single-leg squats until the angle at the knee joint was 30°. After the targeted angle was defined, the participants were asked to find the targeted angle. The difference between the targeted angle and the angle reached by the participants was calculated.

**Results:**

A statistically significant difference in the active joint position sense was found among the ACLR extremity, uninvolved extremity, and control extremity (p < 0.05). The proprioceptive sense between the two extremities in the ACLR group was similar, and the proprioceptive sense was worse than that of the control group.

**Conclusion:**

To our knowledge, this is the first study to evaluate closed kinetic chain position in patients who underwent ACLR, and it showed that proprioceptive sense was still poor in patients with ACLR compared with the control group, even if an average of 24 months have elapsed since surgery.

## 1. Introduction

The anterior cruciate ligament (ACL) is a structure with a dense connective tissue composition that plays an important role in joint stability [1,2]. ACL injuries are the most commonly encountered knee injuries and sports-related lower extremity injuries [3,4]. These injuries cause a reduction or loss of activity that may threaten the careers of athletes [5].

ACL reconstruction (ACLR) is performed in participants with symptomatic instability to restore the function required to return to the preinjury level of activity [5]. Several studies have reported that the following symptoms may be observed after ACLR: pain [6,7], movement limitations [7,8], muscle strength loss [3,9], atrophy [10], balance problems [11,12], and functional deficiencies [10,13]. Proprioceptive losses are also one of the problems encountered after ACLR [10, 11]. Proprioception is defined as the perception of various movements and positions of body parts [14], and it is an important component of neuromuscular performance [15]. Control of movement is the foundation of balance and joint stability. For this reason, proprioception is necessary in the performance of daily living activities, walking, and sports activities [16].

The proprioceptive sense in the knee joint is affected by central and peripheral mechanisms such as articular, cutaneous, and ACL receptors along with the muscles and tendons [15,17]. Moreover, 1%–2% of the ACL volume consists of mechanoreceptors that provide proprioceptive information. A positive relationship exists between the mechanoreceptors found in the ACL and proprioceptive sense. An increase in the mechanoreceptor activation has shown to increase the sense of knee proprioception, thus increasing the functionality of the knee [18,19]. ACL injuries lead to the loss of mechanoreceptors in the joints and a decrease in sensory input, thereby inducing an alteration of the afferent input provided to the central nervous system, affecting sensitivity, impairing motor decision-making, causing inhibition of the muscle motor neurons around the joint, and altering the motor control of the lower extremity [20,21]. A literature review shows that insufficient sensory feedback from the mechanoreceptors of a torn ACL causes disorders in the joints and movement biomechanics further leading to the loss of proprioception [10]. However, information regarding whether proprioception improves after surgery or remains at the postinjury level is unclear [22,23].

Although many studies have evaluated the long-term proprioception of subjects who underwent ACLR [11,17,20,22], to our knowledge, no study has evaluated proprioception using the closed kinetic chain position. In recent years, an increasing number of researchers have recommended that proprioception should be evaluated by using weight-bearing tests [24]. Weight-bearing tests were believed to be more functional and can assess all cutaneous, joint, and muscle proprioceptors that are active during normal daily activities [25,26]. Additionally, various angles have been used to evaluate the proprioceptive sense. In these studies, the targeted angle was usually 45°– 60° [20]. However, knee flexion at 20°–40° is strongly associated with proprioceptive feedback during normal walking [27]. Furthermore, anterior translational force, especially at 20°–30° of flexion, may be the most fatal isolated force associated with ACL injuries [28]. Based on this information, we believe that measurements performed at an angle between these values are more meaningful in terms of functionality. Therefore, this study evaluated proprioception and position to differentiate it from other related studies. This study aimed to evaluate proprioception in sedentary participants who underwent ACL surgery in the closed kinetic chain position in comparison with healthy controls.

## 2. Materials and methods

### 2.1. Participants

Prior to the study, a statistical power analysis was performed for sample size estimation based on previous studies [29]. With an alpha of 0.05 and power of 0.99, the projected sample size (GPower 3.1) was approximately 28 for group comparisons based on previous studies. Thirty-four patients who underwent ACLR using a hamstring tendon graft (time elapsed since surgery = 23.97 ± 15.04 months) and 31 healthy participants whose physical activity level was similar to that of the participants of the ACLR surgery group were included (Table 1). Among healthy participants who agreed to participate voluntarily, those with a physical activity level similar to the participants in the ACLR group, which was assessed according to the short form of the International Physical Activity Questionnaire (IPAQ), were included as healthy controls.

**Table 1:** Demographic characteristics of participants.

		ACLR group (n : 34)	Control group (n : 31)	p
Age (years) (mean ± SD)		29.18 ± 8.16	27.35 ± 5.74	0.299
Body weight (kg) (mean ± SD)		82.54 ± 12.84	77.50 ± 11.78	0.118
Height (cm) (mean ± SD)		176.29 ± 7.64	176.71 ± 7.15	0.895
BMI (kg / cm2) (mean ± SD)		26.58 ± 4.02	24.76 ± 2.98	0.069
IPAQ (MET) (median / IQR)		1346.25 (676 / 2580)	1635.00 (495 / 2826)	0.674
Dominant side (n)	Right	26	31	0.005*
	Left	8	0	

*p < 0.05, ACLR: anterior cruciate ligament reconstruction, IPAQ: International Physical Activity Questionnaire, MET: metabolic equivalent, SD: standard deviation, IQR: interquartile range.

The ACLR group consisted of subjects aged 18–45 years who underwent ACLR surgery with hamstring tendon autograft at least 6 months ago and who have not had an injury for at least 6 months on both extremities. Those with an accompanying posterior cruciate ligament, meniscus, lateral collateral ligament, or medial collateral ligament injury, history of surgery on either lower extremity or a revision surgery, or those who have any systemic or neurological problem were not included in the study.

All participants signed an informed consent form. Ethics committee permission was obtained from the ethics committee of the university (Decision no. 604, dated 25.12.2017).

### 2.2. Procedure

With regard to obtaining a standard proprioceptive input throughout the measurements, all participants wore comfortable shoes and shorts. All measurements were performed by the same researcher. All measurements were started with the uninvolved extremity in the ACLR group and the dominant extremity in the control group. The dominant extremity was determined by questioning which foot is used for kicking a ball. It was repeated on the other extremity then.

The participants’ age, body weight, height, and body mass index (BMI) were recorded. Additionally, the site of injured extremity, date of injury and surgery, history of musculoskeletal injuries, treatment received after surgery, and, if applicable, duration of physiotherapy were noted by questioning the subjects who had undergone ACLR.

The participants’ level of physical activity was assessed using the IPAQ short form. The questionnaire consists of seven questions that assessed the time spent and the frequency of activities in four intensity levels of sitting, walking, moderately severe activities, and rigorous activities in the last 7 days. Physical activity was expressed as weekly total metabolic equivalent (MET minutes/week) [30,31].

Proprioception was assessed with an active angle repetition test, using Dualer IQ Digital Inclinometer (J-Tech Medical, Midvale, UT, USA), in the closed kinetic chain position while standing. One part of the inclinometer was placed on the lower one-third section of the lateral face of the femur along the joint line with a strap. The other part of the inclinometer was placed on the lower one-third lateral section of the leg along the joint line (Figure). The test was initiated with the knee in the extension position, and the participants were asked to perform single-leg squats until the targeted angle was reached. Participants were allowed to support themselves with one hand to prevent loss of balance while performing single-leg squats. When they reached the targeted angle, which was 30°, they were asked to stop and maintain this position for 5 s. Then, they were told to return to the starting position (full knee extension). After the targeted angle position was defined three times, the participants were asked to find the targeted angle as accurately as possible in three attempts [32]. The difference between the targeted angle and the angle achieved by the participant was recorded as an absolute angular error. The relative angular error (RAE) was calculated by taking the arithmetic average of the difference between the targeted angle and the angle achieved by the participant [16].

**Figure 1:**
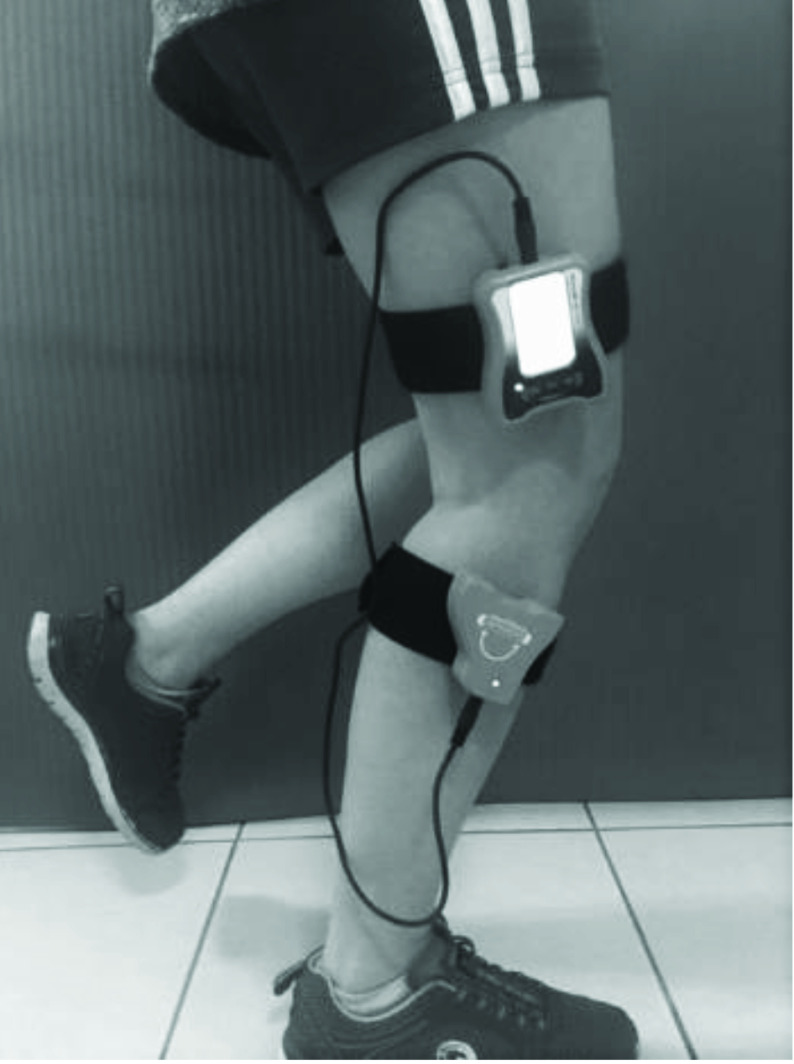
The measurement of proprioception in functional position.

RAE = | (targeted angle − 1st trial) | + | (targeted angle − 2nd trial) | + | (targeted angle − 3rd trial) | / 3 [15,32].

### 2.3. Statistical analysis

Statistical analysis was performed using SPSS Version 22.0 (SPSS Inc., Chicago, IL). Data normality was analyzed using analytical methods (i.e. the Kolmogorov–Smirnov/Shapiro–Wilk test). For the descriptive analysis, parametric data were expressed as mean ± standard deviation and nonparametric data as median ± IQR. For the analysis, the presence of a difference between the right and left lower extremities of the control groups was initially verified. The Wilcoxon test was used for the analysis of nonparametric data. As no significant difference was found between the two lower extremities of the control groups, the lower extremity of the ACLR group was matched with the lower extremity of the control group. The Kruskal–Wallis test was used to compare nonparametric data among the ACLR extremity, uninvolved extremity, and matched extremity of the control group. The significance level was set at 0.05. The Mann–Whitney U test was used for analysis of pairwise comparisons. Bonferroni correction was applied and p significance value to be used for pairwise comparisons was determined as 0.017.

## 3. Results

Participants’ demographic information, activity level, and dominant sides are presented in Table 1. While there is a statistically significant difference between the groups in terms of the dominant side, no statistically significant difference was found in age, body weight, height, BMI, and activity levels between the ACLR group and the control group. 

A mean of 23.97 ± 15.04 months had elapsed since the individuals had undergone ACLR surgery (range, 9–40 months). Moreover, 21 (61.8%) patients received rehabilitation after surgery and 13 (38.2%) did not receive rehabilitation. At a median 4 (range, 0–60) weeks rehabilitation was received. Of the individuals who underwent ACLR, 18 had undergone surgery on their right knees and 16 on their left knees.

As regards the result of active joint position sense, a statistically significant difference was found among the ACLR extremity, uninvolved extremity, and matched extremity of the control group (Table 2). The proprioceptive sense in the ACLR extremity and uninvolved extremity was similar (p = 0.699); however, the proprioceptive sense was worse than that of the matched extremity of the control group (p < 0.001).

**Table 2:** The comparison of ACLR extremity, uninvolved extremity with and control groups extremity in terms of proprioception

	ACLR extremity(mean ± SD)	Uninvolved extremity (mean ± SD)	Control groups extremity(mean ± SD)	p
Active position sense (°)	4.41 ± 2.55	4.17 ± 2.72	2.00 ± 1.59	0.000 * ¥ Ф

ACLR: anterior cruciate ligament reconstruction, SD: standard deviation

## 4. Discussion

In this study, proprioception was assessed in patients who underwent ACL surgery in the closed kinetic chain position. It was found that the proprioceptive sense in these patients, both in the ACLR extremity and uninvolved extremity, was worse than that of the matched extremity of the control group.

Studies have stated that deficits can occur in both injured and contralateral extremity after ACL injuries. Therefore, the results should be compared with a control group matched for age, sex, and physical activity level [33]. Participants who maintain their strength and flexibility with regular physical activity can perform their daily activities easily and they have superior physical fitness [34]. In our study, the control group comprised participants who had the same physical activity level and characteristics as the participants who underwent ACL surgery. Thus, the positive effects that physical activity could particularly induce were excluded.

Proprioception can be evaluated in various positions; however, the evaluation of joint position sense in the nonweight-bearing position is not functional [35]. Weight-bearing positions were reported to provide better information about the joint position sense, which leads to acquisition of more accurate and reliable results [24]. Weight-bearing positions cause a higher joint reaction force and greater muscle co-contraction during activities than nonweight-bearing positions. During weight transfer, sensorimotor function increases because of the increase in the muscle activity and inputs from the joints. Hence, weight-bearing positions may provide better information about the afferent feedback during functional activities [35]. In the closed kinetic chain position, more joint receptors, Golgi tendon organs, and muscle spindles are stimulated. Researchers have also stated that the closed kinetic chain position provides a more accurate reflection of the joint position sense. Additionally, it provides better information about proprioceptive acuity or motor control. The closed kinetic chain positions are more functional and similar to position injuries; therefore, these positions will reveal deficits in a better way [36]. Suner-Keklik et al. investigated 22 healthy individuals and showed that the measurement of proprioception in the closed kinetic chain position with the same inclinometer was valid and reliable [32]. For this reason, the proprioceptive sense was evaluated in the closed kinetic chain position in our study. The present study revealed that the proprioceptive sense was similar between the two lower extremities, and when compared with the control group, the proprioceptive sense was worse in the extremity that underwent ACLR than in the uninvolved extremity. This observation may be attributed to the occurrence of a proprioceptive deficit, similar to that of the extremity that underwent ACL surgery, in the uninvolved extremity due to crossover inhibition. Alternatively, after surgery, individuals may have restricted their movement due to the fear of reinjury, which may have decreased the input to the extremities, eventually leading to proprioceptive deficits.

To our knowledge, no studies have evaluated proprioception in the closed kinetic chain position. However, some studies have evaluated proprioception in the open kinetic chain position after ACLR. A study that evaluated the joint position sense by using an isokinetic dynamometer at the targeted angles of 30°, 45°, and 75° in individuals with chronic disease who underwent ACL surgery presented no difference in the joint position sense between the two extremities [37]. That study supports our study in terms of the similar proprioceptive senses of the two extremities. On the contrary, Katayama et al. presented different results. They assessed proprioception with an isokinetic system in patients with isolated ACL rupture and found that patients had decreased proprioception in the injured extremity compared with the contralateral extremity [38]. Although the closed kinetic chain position was not used as an evaluation method in these studies, these studies do not have a control group. A metaanalysis revealed that subjects who underwent ACL surgery had better proprioception than individuals with unrepaired ACL injuries. However, these differences exist because either healthy individuals or individuals with uninjured extremity were used as a control group. For this reason, in future studies, researchers recommended that the results of both individuals who underwent ACLR surgery and individuals who did not undergo surgery should be compared with the results of a control group of healthy individuals [39]. Several studies have evaluated proprioception after ACLR with this setup. Relph et al. examined proprioception in the sitting position in patients who underwent ACL surgery in the chronic phase. Similar to our study, they found that proprioceptive deficit was more significant in the extremity of patients who underwent surgery than those with both uninjured extremities and healthy controls [22]. A study that evaluated proprioception in a sitting position in patients with ACL injuries and healthy subjects found that the injured extremity and contralateral extremity had similar proprioceptive deficits, and the proprioceptive deficits were greater in patients with ACL injuries than in the control individuals [40]. In a study that assessed subjects who underwent ACL surgery and healthy controls, proprioception was assessed in the supine position. Contrary to our study, that study found that none of the individuals had proprioceptive deficits [20]. The contradicting results may be related to the use of different evaluation methods for proprioception.

A review also revealed that proprioceptive deficits in both injured and noninjured extremities are encountered in unilateral ACL rupture [17]. This observation is attributed to the fact that a portion of the mechanoreceptors in that region are damaged after an injury or surgery. These receptors do not regenerate; however, individuals can adapt to the altered proprioceptive feedback and, thus, maintain the levels of physical activity. For this reason, exercises aimed at improving proprioception after injury are performed in clinics [20].

The examination of the rehabilitation status and treatment duration in the ACLR group revealed that some individuals received short-term rehabilitation and some had none at all. This may have led to the persistence of proprioceptive deficits despite the passage of time since surgery. In this study, the factors that caused proprioceptive deficits could not be identified because the participants were not included in a standard rehabilitation program after surgery.

In conclusion, we believe that our study is the first to evaluate proprioception after ACL surgery in the closed kinetic chain position. Our results showed that the persistence of proprioceptive deficits even after a long time since surgery and the injured extremity could not reach the proprioception level in the matched leg of the healthy control. Further studies should be conducted with subjects who are enrolled in similar rehabilitation programs for a longer duration. Thus, changes over time in proprioception after surgery will be clearly evaluated.

## Informed consent

All participants signed an informed consent form. Ethics committee permission was obtained from Gazi University Non-Interventional Clinical Research Ethics Committee with the decision dated 25.12.2017 and numbered 604.
